# Quantification of protein and latex allergen content of various natural rubber latex products 

**DOI:** 10.5414/ALX01461E

**Published:** 2017-08-04

**Authors:** Y. von der Gathen, I. Sander, A. Flagge, T. Brüning, M. Raulf-Heimsoth

**Affiliations:** Institut für Prävention und Arbeitsmedizin der Deutschen Gesetzlichen Unfallversicherung, Institut der Ruhr-Universität Bochum (IPA), Bochum, Germany

**Keywords:** latex allergy, allergen content, examination gloves, household articles, immunoassay, single allergen detection

## Abstract

Introduction: The use of natural rubber latex (NRL) products can cause IgE-mediated allergic reactions in exposed people. The aim of this study was to quantify the content of protein and latex allergens of currently available NRL products to estimate the allergenic potential of these products. Methods: 14 household articles (pacifiers, baby bottle nipples, condoms, household and disposable gloves, toy balloons, and Band-Aids) as well as 18 NRL examination gloves currently used by healthcare workers were investigated. Extracts of the examination gloves were prepared according to the standard method DIN EN 455-3, which contains requirements and testing for biological evaluation of single use medical gloves. The protein content was determined with a modified Lowry method. Latex allergen content was measured using an IgE-inhibition immunoassay with a mix of serum-sensitized patients as detection antibody sources and the latex ImmunoCAP as solid phase. The allergens Hev b 1, 3, 5, and 6.02 were determined using available immunoassays. Results: In 5 out of 18 examination gloves, the protein content was under the detection limit. The other 13 gloves contained protein between 7.1 and 92.3 µg protein/g material. Five glove brands contained protein concentrations above the recommended reference value of 30 µg protein/g material. Latex allergen could be measured in 12 out of 18 NRL gloves. In only 3 gloves could none of the allergens Hev b 1, 3, 5, and 6.02 be detected. Protein and Hev b 1 could be measured in the examined childcare products, while the concentrations of the latex allergens Hev b 3, 5, and 6.02 were mostly under the detection limit. Boiling of childcare products led to a reduction of protein and allergen content. In some of the other daily-used NRL articles, the protein and allergen contents were even higher than in gloves. Conclusion: Our study demonstrated that protein, and particularly latex allergens, were detectable in currently available examination gloves as well as in household articles whereby a risk for sensitization and/or induction of allergic symptoms could not be excluded.

German version published in Allergologie, Vol. 35, No. 6/2012, pp. 310-322

## Introduction 

The allergenic components in products made of natural rubber latex (NRL), which can induce IgE-mediated type I allergies and symptoms in exposed people, are proteins of the milk of the rubber tree, *Hevea brasiliensis [* 31]. So far, 17 NRL allergens and isoforms with a molecular weight of 4.7 – 60 kD have been described: Hev b 1 – 14 [22]. 

With the increase of viral infections, mainly HIV and hepatitis, in the 1980s, there was a surge in the use of NRL gloves. More and more powdered NRL gloves were used in the healthcare sector and, among other things, the increased production led to a reduced quality of the gloves in terms of an increased protein content [13, 18]. The proteins bound to the powder were, e.g., released to the room air when the gloves were put on or off. People would inhale these proteins and in some allergic reactions resulted [7, 9, 21, 33]. 

The reduction of latex sensitization can be attributed to findings on the allergenicity of latex products, the importance of the powder as a carrier of latex allergens, as well as to the implementation of numerous effective prevention measures [1, 2, 16, 31, 34]. 

Major allergens for healthcare professionals and patients with spina bifida were identified with the help of recombinant single allergens. The major allergens for healthcare professionals are considered to be Hev b 2, 5, 6.01, and 13, while Hev b 1, 2, 3, 5, 7, and 13 are considered to be the major allergens for patients with spina bifida [10, 14, 15, 30, 35]. 

As latex allergy is incurable and can be very severe [11], investigation on latex allergy is still necessary to avoid new sensitizations and to be able to contain the severity of existing allergies. In their study, Crippa et al. [17] concluded that all products made of NRL should be labeled with “contains natural rubber latex” and a warning that allergic reactions could be caused. 

Furthermore, numerous products for everyday use are still made of NRL [6], so that also in people’s homes, sensitization against this material cannot be excluded. Thus, it is helpful to assess the sensitizing potential of currently available latex products. 

The aim of our study was to assess the allergenic potential of currently available latex products. To do so, we analyzed the protein and latex content of 14 latex products used at home (pacifiers, baby bottle nipples, condoms, household and disposable gloves, toy balloons, and Band-Aids) and 18 latex examination gloves used by healthcare workers. The protein and allergen content of these products was determined. In addition, the major allergens Hev b 1, Hev b 3, Hev b 5, and Hev b 6.02 were quantified using commercially available ELISAs (FITkits) or a special Hev b 1-ELISA developed at our institute (IPA). 

## Methods 

### Material 

A total of 18 different powder-free examination and surgical gloves (G, status 2009) were investigated: 2 – 4 different models from 6 different manufacturers (Ansell, Augustus, Hartmann, Mölnlycke, Rösner-Mautby, and Unigloves) and 1 double-glove system. In addition, several products of daily use (PDU) that can come into contact with skin or mucosa and that are available at German drugstores or toyshops were analyzed: condoms (Billy Boy, chaps, Durex, and Ritex), pacifiers and baby bottle nipples (babylove, nip, and NUK), Band-Aids (das gesunde Plus), household and disposable gloves (both Profissimo), and toy balloons (Adic B.V.). As a positive control (G02), we used a powdered glove with a known protein and allergen content, which is used for provocation tests in occupational situations (manufactured by Unigloves). A glove manufactured by Yulex and made of guayule (Parthenium argentatum) rubber was used as a negative control (G01). 

### Extraction of latex material 

Extraction was carried out using two methods. 

***Extraction according to the European standard DIN EN 455-3 [19]***

**Figure 1.**
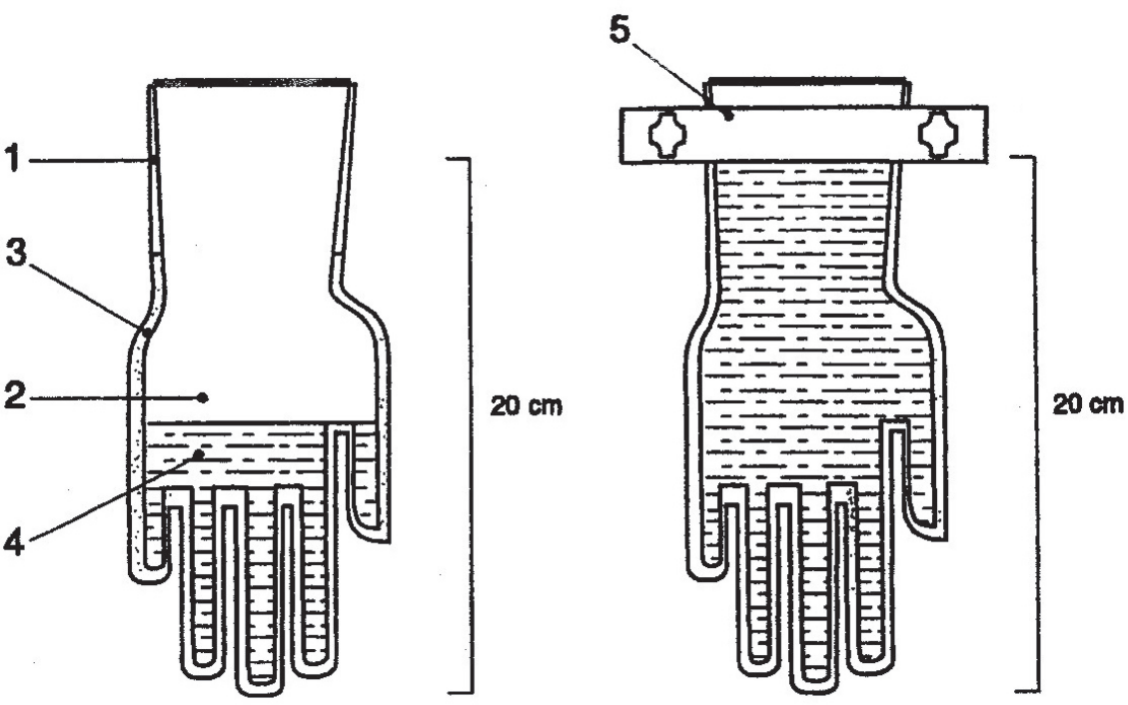
Extraction method for gloves according to the European standard DIN EN 455-3 [19]. 1: outer glove; 2: inner glove; 3: extraction buffer; 4: dye solution; 5: glove clip. Source: DIN EN 455-3 (1999, German version) [19].

One pair of gloves was needed for each extract. A mark was made on the outer glove, 20 cm from the tip of the middle finger, and the glove was weighed. The rest of the procedure is shown in Figure 1. Before the gloves were closed, the air bubbles were removed; subsequently, the gloves were shaken on a horizontal shaker (200/min) for 2 hours. If the extract was not stained blue, it was centrifuged at 3,600 × at room temperature (RT) for 15 minutes. The supernatant was filtered through a 0.22 µm filter, portioned, and stored at –70 °C. The gauntlet of the outer glove was cut off at the 20-cm mark, and the (dry) weight was determined. The weight of the extracted part of the glove was determined by subtracting the weight of the gauntlet from the total weight. 

***Extraction according to a procedure developed at our institute (IPA method)***

Extraction of the gloves was carried out according to Baur et al. [8], with minor modifications. 3 g of each latex product were cut into little pieces (~ 1 cm²; pacifiers and baby bottle nipples ~ 0.1 cm²), 20 mL of extract solution (phosphate-buffered saline solution (PBS)) were added, and then these mixtures were shaken at a high level in a water bath at 37 °C for 2 hours (and additionally mixed in a vortex mixer every 15 minutes). Larger pieces of latex were removed and the extract solution was centrifuged (3,600 × g, RT, 15 minutes). The supernatant was filtered (0.22 µm), portioned into 1 mL samples, and stored at –70 °C. 

The extraction of the products of daily use was also carried out according to this method. 

### Boiling of baby articles 

As according to the manufacturers’ instructions, baby articles, like pacifiers or baby bottle nipples, should be boiled before use; we also wanted to investigate the effect of this boiling. With this aim, the baby articles were put into boiling water for 5 minutes and dried thereafter. 

### Determination of protein content 

The protein content of the gloves and products of daily use was determined according to the DIN EN 455-3 standard, using a modified Lowry method employing an ovalbumin standard (as recommended by the American Society for Testing and Materials [3]). 

### Determination of the latex content using IgE inhibition testing 

The latex content was determined by IgE inhibition testing [8] using the ImmunoCAP system (Phadia, Uppsala, Sweden; k82 without rHev b 5 spike [25, 29]). A serum pool of 12 serums of latex-sensitized patients was used as an antibody source. As a latex standard for the inhibition experiment, particle proteins of latex milk stabilized with 0.2% ammonia at a concentration of 0.1 – 20 µg allergen/mL were used. A sample with only a dilution buffer was used as a control. For determination, 40 µL of pooled serum were added to 20 µL of the inhibitor; measurement was carried out using the ImmunoCAP system. The linear measuring range of the standard curve was between 20% and 83%. The mean detection limit was 0.2 µg/mL. 

### Determination of the latex allergen Hev b 1 

This sandwich ELISA is based on two different monoclonal antibodies against the latex allergen Hev b 1 (modification of the assay described by Raulf-Heimsoth et al. [32]). The capture antibody (II4F) detects the amino acids 46 – 54, the detection antibody (II4G9) detects the amino acids 122 – 134 of the protein Hev b 1. Between all incubations (100 µL each), washing with 3-times 250 µL PBST (PBS with 0.05% (v/v) Tween 20) were carried out. The wells of the MaxiSorp microtiter plates with increased surface (Roskilde, Denmark) were coated with capture antibody in a 1 : 400 dilution over night at a temperature of 4 °C. The wells were blocked with 2% BSA (w/v) in PBS for 2 hours at room temperature. The standard was applied with concentrations between 2 ng/mL and 250 ng/mL and samples in various dilutions (1 hour, room temperature). After 1.5 hours of incubation with the detection antibody (dilution 1 : 1,500) at room temperature, streptavidin poly-horseradish peroxidase 80 (Fitzgerald, Concord, MA, USA; 1 : 20,000) was added for 1 hour. 2,2’-azinobis 3-ethylbenzthiazolin-6-sulfonic acid manufactured by Sigma (Steinheim, Germany) was used as a substrate. The mean measurement range of the Hev b 1-ELISA was between 15 ng/mL and 228 ng/mL; the detection limits of 7 test runs were used for the calculation. 

### Determination of the latex allergens Hev b 3, 5, and 6.02 

The single allergens Hev b 3, Hev b 5, and Hev b 6.02 were quantified using the commercially available immunological test (FITkit) manufactured by Quattromed (Tartu, Estonia). 

The calculations of the detection limits are based on 3 (Hev b 3-FITkit, Hev b 5-FITkit) or 4 (Hev b 6.02-FITkit) test runs. The mean detection limits were 15 ng/mL (Hev b 3), 7 ng/mL (Hev b 5), and 5 ng/mL (Hev b 6.02), respectively. 

### Evaluation and statistics 

The software SoftMax Pro 4.7.1 (Molecular Devices, Sunnyvale, CA, USA) was used to determine the proteins and single allergens and to calculate the values. To calculate the Pearson and Spearman correlation coefficients as well as the significance of the correlation (p < 0,05) and the Bland-Altman diagrams, the software program Prism 5.01 (GraphPad, San Diego, CA, USA) was used. Values below the detection limit entered the calculation of the correlation coefficients as 2/3 the detection limit. For the Bland-Altman analysis only measurable results were used. 

## Results 

### Protein and latex allergen content of gloves 

Extracts of 20 different examination and surgical gloves were prepared according to the standard method DIN EN 455-3. 18 of these gloves (G03-G19) could be commercially obtained in Germany in 2009 (Table 1). The protein content of 13 of these gloves was above the detection limit within a range between 7.1 and 92.3 µg/g (Table 1). 

For 12 of these 18 gloves, the latex allergen content could be determined and was between 0.9 and 15 µg/g glove (Table 1). In 3 gloves (G04, G12, and G16), the content of latex allergen was > 10 µg/g and thus markedly higher than in the other 9 gloves (maximum value of these: 2.7 µg/g). 

The Hev b 1 content could be determined in 14 cases. Most values were within the range of 50 – 200 ng Hev b 1/g glove. Exceptions were G04, G12, and G16 with contents of almost 1,000 – 2,566 ng Hev b 1/g glove. Hev b 3 could be quantified in 5 of the 18 gloves (Table 1), with the highest value being 2,271 ng Hev b 3/g glove (G04). Hev b 5 was also quantified using FITkit and was measurable in 10 of the 18 gloves. The highest Hev b content was ~ 1,000 ng Hev b 5/g material (Table 1, G04). In 4 gloves (G04, G12, G16, and G18), the Hev b 5 content was markedly higher than in the other gloves. Of the 18 gloves examined, the Hev b 6.02 content could be determined in 7 (55.4 – 1,936 ng/g). The by far highest quantifiable value was 1,936.8 ng Hev b 6.02/g glove (G16). All other gloves had markedly lower Hev b 6.02 contents (Table 1). The glove G04 had the highest latex allergen content as well as the highest contents of the 3 single allergens Hev b 1, 3, and 5. The single allergen Hev b 6.02 could not be detected in the extract of this glove. 

### Comparison of the extract methods for gloves 

**Figure 2.**
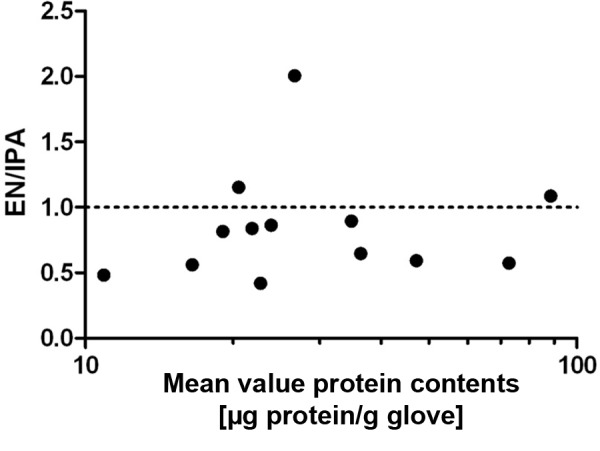
Bland-Altman plot comparison of protein determination in extracted gloves according to EN standard and our method (IPA method). The mean values of the protein contents of the EN and IPA methods were entered against the quotient (EN/IPA) of these values. The dashed line represents the quotient that would result from identical values of both extraction methods.

Besides the DIN EN 455-3 method, we also prepared additional extracts according to an approach developed at our institute (IPA). The latter is easier to carry out, so that it is interesting to see if the results are comparable. The protein contents measured with the IPA or the EN method correlated well, with a correlation coefficient of r = 0.88 (n = 18). In the Bland-Altman analysis (Figure 2), the mean value of the ratios was 0.84 (n = 13), with a standard deviation of 0.42. In 10 of 13 cases, the IPA method was able to extract more protein. Only in 1 case was, the protein content approximately twice as high in the extract prepared with the DIN method than in the one done with the IPA method. 

**Figure 3.**
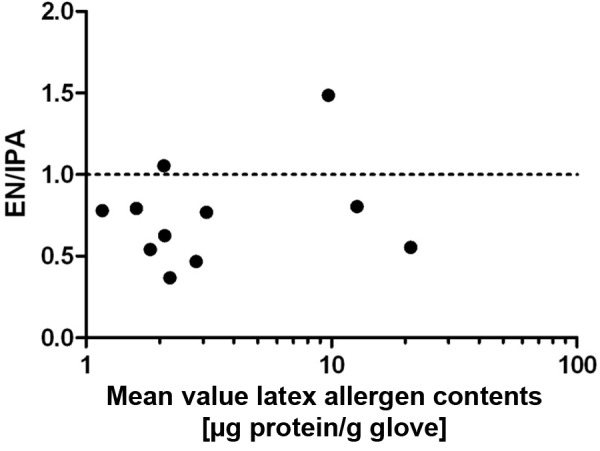
Bland-Altman plot comparison of latex allergen content as measured by IgE inhibition test in extracted gloves according to EN standard and our method (IPA method). The mean values of the latex allergen contents of the EN and IPA methods were entered against the quotient (EN/IPA) of these values. The dashed line represents the quotient that would result from identical values of both extraction methods.

Generally, the latex allergen contents determined by IgE inhibition were also higher when the extracts were prepared using the IPA method (Figure 3). The mean value of the EN/IPA ratio was 0.75, with a standard deviation of 0,31. The mean values of the latex allergen contents of the IPA and EN methods correlated significantly (r = 0.92). 

### Protein and latex allergen content of products of daily use 

The protein concentration of various products of daily use could be determined in 13 of 14 extracts. The values were between 15.3 and 202.9 µg protein/g material (Table 2). 

The latex allergen content could only be determined in 8 of the 14 products using IgE inhibition testing. In only 1 pacifier, was the latex allergen content marginally measurable, and no latex allergen content could be detected in the other pacifiers/baby bottle nipples. Also, in one condom (Durex, PDU09), no latex allergen could be found. In all other products of daily use, latex allergen could be determined (values of up to 4.4 µg allergen/g material). However, the content of latex allergen in the toy balloon (PDU11) was markedly higher: 21.5 µg allergen/g material. 

Hev b 1 could be quantified in 12 of the 14 products. Most products, including baby products, had a Hev b 1 content of ~ 200 – 2,000 ng/g. In 3 of the 4 tested condoms (PDU07, PDU08, and PDU10), the Hev b 1 contents were markedly higher, with the condom PDU08 having the highest Hev b 1 content of all tested products: 14.35 µg Hev b 1/g material. Only in the disposable gloves and in the Band-Aids, could no Hev b 1 be detected. Hev b 3 could be quantified in 2 products of daily use (condoms PDU07 and PDU08), with the condom PDU08 having the higher Hev b 3 content. 820 ng/g material (Table 2). Four of the 14 products of daily use contained Hev b 5. In all the pacifiers and baby bottle nipples, the Hev b 5 content was below the detection limit. Of the condoms, the Hev b 5 content could only be quantified in one (PDU10). The highest Hev b 5 content of 3,125 ng/g material was detectable in the toy balloon (PDU11). Hev b 5 was also detected in the disposable and household gloves (Table 2). Hev b 6.02 could be demonstrated in 7 products of daily use. No Hev b 6.02 was detectable in the pacifiers, baby bottle nipples, or in the condom PDU09 (Table 2). The highest value of 8,000 ng Hev b 6.02/g material was measured in the toy balloon, while the contents of the other products were between 100 and 600 ng Hev b 6.02/g material. 

### Impact of pre-treatment on the protein and latex allergen content of baby products 

In 5 pacifiers and baby bottle nipples (PDU01, PDU03 – PDU06), only proteins and Hev b 1 could be quantified (Table 3). Latex allergen content could additionally only be detected in 1 not pre-treated pacifier (PDU02), while the allergens Hev b 3, 5, and 6.02 were not quantifiable. The protein content of the not pre-treated pacifiers/baby bottle nipples was between 15 µg and 127 µg protein/g material, while the content after boiling was below 50 µg/g. In the 3 pacifiers/baby bottle nipples with the highest protein content (PDU01, PDU02, PDU05), proteins were still measurable after boiling. However, the values were far below those measured in the untreated products. Boiling, which should be carried out before the first use, led to an approximately 80% reduction of protein content. In the pacifiers (PDU02), no latex allergen was detected after boiling (Table 3). Boiling was able to reduce the content of Hev b 1 (this single allergen was detectable in all pacifiers/bottle nipples) by 16 – 79%. 

## Discussion 

### Protein and latex allergen content of gloves 

While in the 1990s about 10% of healthcare professionals were affected by latex allergy, the number of suspected cases of occupational latex allergy has been decreasing since 1999. This could be achieved thanks to numerous effective preventive measures [2]. For example, since 1998, powdered latex gloves have had to be replaced. The German technical standards for hazardous material (TRGS 540) recommend that latex gloves should be powder-free and contain only few proteins [34]. The protein content should be below 30 µg protein/g glove, otherwise the employees would have to be examined by the company physician (TRGS 406) [34]. The European standard DIN EN 455-3 defines the examination and biological evaluation of medical gloves as well as the extraction methods for the determination of the protein content [19]. Based on these requirements, we prepared extracts from 18 commercially available gloves in 2009. In 5 of these 18 glove extracts, the protein content was below the detection limit, and in 8, it was below 30 µg/g glove. These 13 gloves could be used in healthcare institutions and laboratories without examinations by the company physician being necessary. In 5 gloves, the protein content was above 30 µg/g, and 1 glove (G16) even contained 3-times the reference value. 

As not all proteins are necessarily allergens, we not only evaluated the protein content but also the content of latex allergen. Values of up to 15 µg/g were measured. Of the 5 gloves with high protein content, 3 had the highest latex allergen content. In the other 2, no latex allergen was detected. This suggests that a higher protein content does not have to mean a higher latex allergen content. Nevertheless, the determination of the protein content and of the total latex allergen content showed a relatively good, significant correlation according to Spearman (r = 0.651; p < 0.05). Similar results were obtained by Audo et al. [4] who measured, among other things, the latex allergen content using IgE-ELISA inhibition and compared it with the protein content (modified Lowry method); the total Spearman correlation for all 98 gloves was r = 0.78 (p < 0.001). In 1997, Baur et al. [8] investigated the protein and latex allergen content of 62 latex-containing gloves and of other products (e.g., catheter, latex mattress, and so forth). No significant correlation between the measurable protein/latex allergen contents and the investigated latex products could be demonstrated (r = 0.40). 

Peixinho et al. [28] examined 41 brands of gloves used at Portuguese healthcare institutions in 2006. They determined the single allergen contents of Hev b 1, Hev b 3, Hev b 5, and Hev b 6.02 with the commercially available FITkits. No Hev b 1 could be detected in 11 gloves, and in 20 gloves only very low concentrations (0.05 µg/g) were detected. In contrast, we were able to quantify Hev b 1 in 14 of 18 samples (78%) using the Hev b 1 ELISA developed at our institute. In the investigation by Peixinho et al.as well as in our own, the allergen Hev b 3 was detected in 46% of all samples and only in 28% of the glove samples. Hev b 5 and Hev b 6.02 was measurable in all gloves in their study, while these 2 allergens could be quantified in only 26% and 39% of our glove samples, respectively. Thus, Hev b 5 and Hev b 6.02 were more frequently detected than Hev b 3. Palosuo et al. [27] also used the commercially available ELISA (FITkit) to quantify the allergens Hev b 1, 3, 5, and 6.02 in gloves. They examined 208 brands of medical gloves available in Finland in 1999, 2001, and 2003 and compared the sums of the 4 single allergens with the results of an ELISA inhibition test based on human IgE, with a high Spearman correlation resulting (r = 0.87). In our investigation, we also compared the sums of the results of the Hev b 1 ELISA and the 3 FITkits with the results of the IgE inhibition test and calculated a significant correlation according to Spearman (r = 0.843; p < 0.05). 

Comparing the protein content with the sum of single allergens, a significant correlation according to Pearson (r = 0.746; p < 0.05) as well as according to Spearman (r = 0.787; p < 0.05) resulted. Three of the gloves with the highest protein and latex allergen content also had the highest sums of single allergen content. The sums of the determined single allergen concentrations of these gloves were up to almost 6,000 ng/g glove and thus markedly above the contents of the other gloves (< 1,000 ng/g). In the gloves with the highest Hev b 1 contents, these were up to 60-times higher than in the glove with the lowest Hev b 1 content. The allergen profiles show that not only Hev b 1 but also the other 3 single allergens could be detected in many gloves. Although Hev b 1 was shown most frequently, its concentration was not always the highest. In some cases, also Hev b 3, 5, or 6.02 could be predominating. 

As our investigation shows, it is possible to manufacture gloves with a very low allergenic potential. This has to be aimed at by manufacturers. However, the protein content should not be the only parameter to estimate the allergenic potential of gloves because considerable concentrations of single allergens could also be detected in gloves without measurable or only minor amounts of protein. 

### Comparison of the extract methods for gloves 

Comparing the two extract methods (EN method vs. IPA method), we found that with the IPA method it was possible to extract more proteins and latex allergens. While with the EN method the inner and outer part of two gloves are extracted at the same time by nesting one glove into the other, with the IPA method, the gloves are cut into small pieces, which significantly increases the extracted surface. In addition, our IPA method is less complex and extracts a standardized mass of the product (3 g), while the weight of the gloves varies widely when the EN method is used. Although the values obtained were higher when the IPA method was used, good correlations between the IPA and the EN methods were found (protein content: r = 0.88; latex allergen content: r = 0.92). 

### Protein and latex allergen content of products of daily use 

While there are regulations to protect the users of medical gloves, only little is known about the allergenic potential of many latex-containing products of daily use. Thus, we also examined the sensitizing potential of a selection of such products. 

Only in 1 of the 14 products of daily use was the protein content below the detection limit. In almost 86% of extracts from products of daily use (which were prepared using the IPA method), protein contents of > 30 µg/g (recommended reference value for medical gloves) were measured. In the products of daily use, high protein contents were measured more frequently than in gloves, and the protein contents were markedly higher. 

More than half of the products of daily use (8 of 14) contained a quantifiable amount of latex allergen. However, similarly to the gloves, a high protein content did not necessarily reflect a high latex allergen content. Nevertheless, the values correlated significantly (r = 0.665; p < 0.05). 

Lundberg et al. [26] studied medical products, albeit no products of daily use. Over a period of 3 years, they examined the protein and latex allergen content of 92 batches of catheter balloons manufactured by a Swedish company. The protein content was evaluated by the modified Lowry method, the latex allergen content by Latex EIA Assay, ImmunoCAP (Phadia). In contrast to our results, Lundberg et al. found no correlation between the values of the protein determination and the EIA inhibition (r = 0.085). 

In most of the condoms, very high protein and latex allergen contents were detected. Some of the quantified contents of the 4 single allergens were also very high. The extract of the condom PDU08 showed particularly high values in all tests, while in the condom PDU09, only the value measured by Hev b 1 ELISA was high. Docena et al. [20] also measured high protein contents of up to 740 µg/g material (in our investigation up to ~ 200 µg/g material) in condoms by modified Lowry method and demonstrated the presence of various allergenic proteins. Of the other products of daily use, the Band-Aids and the toy balloon had partially very high values, while the values of the disposable and household gloves were in the median range of measured values. 

The toy balloon had a high protein content, and its latex allergen and single allergen contents were particularly high. In addition, many toy balloons are coated with powder to avoid conglutination. Similarly to the gloves, allergens are probably bound to the powder and released to the air together with the powder. In this context, a case reported by Baker and Hourihane in 2008 [5] is interesting: in a boy with spina bifida, his latex allergy had never caused problems because only latex-free examination materials were used; however, he experienced dyspnea and had to be treated in hospital when at his 5th birthday a toy balloon burst and latex dust was released. While powdered gloves will have to be replaced by powder-free, latex allergen-poor, or other adequate gloves (TRGS 540), household products and products of daily use are still insufficiently controlled. 

The most frequently detected single allergen in products of daily use was Hev b 1, and in most products the quantified amounts were relatively high. 

### Impact of pre-treatment on protein and latex allergen content 

Pacifiers and baby bottle nipples should be boiled for 5 minutes before first use, as recommended by the manufacturers. In our study, this reduced the protein content by an average of ~ 80% so that protein contents could no longer be detected, and the highest value was significantly reduced. The reduction of the protein content could be due to the fact that boiling the pacifiers/bottle nipples in water for 5 minutes represents a first extraction, and thus most proteins and allergens had been washed out before we prepared our extract. It would be interesting to examine the water in which the products were boiled to see whether allergens could be found. However, as heating denatures proteins, it is unclear whether, with the usual test procedures, allergens could be detected in the water. 

Boiling was able to reduce the Hev b 1 content by 16 – 79%, which could be demonstrated in untreated as well as in boiled pacifiers/bottle nipples. We did not find an explanation for the large range of Hev b 1 reduction in the boiled pacifiers/bottle nipples. 

## Conclusion 

In this study, we investigated various latex products that were commercially available in Germany in 2009 (gloves, baby products, condoms, Band-Aids, toy balloon). The results suggest that many products contain allergenic latex proteins. Our investigation underlines the large variability of the protein, total latex, and single allergen contents of gloves and products of daily use. This variability is strongly influenced by differences in the composition of raw latex extracts and in the production processes. Among the production processes, important parameters are the number, quality, and temperature of leaching baths. In addition, the quality of natural rubber depends on the use of vulcanization accelerators and preservatives as well as on the storage time of raw materials and finished products [23, 24]. By adding protein-depleting enzymes or by irradiation with gamma-rays for sterilization, the protein content, and thus the allergen content, can be reduced [12]. 

Our results show that it is not only important to control the latex allergen and protein contents of gloves used by healthcare professionals but also to control latex-containing products of daily life. Not only gloves and latex products used by healthcare professionals but also products of everyday life should be produced in a latex-poor form. 

## Annotation 

This manuscript is based on the diploma thesis written by Dipl.-Biol. Yvonne von der Gathen in the field of biology and biotechnology. The detailed results are available as an IPA report at http://www.ipa.ruhr-uni-bochum.de/pdf/10-12-06 IPA_Report.pdf. 

**Table 1.** Protein, latex allergen, and single allergen content of gloves (G) after extraction according to the standard EN 455-3.

Product	Name	Protein content (µg/g)	Latex allergen content (µg/g)	Hev b 1 content (ng/g)	Hev b 3 content (ng/g)	Hev b 5 content (ng/g)	Hev b 6.02 content (ng/g)	Sum of single allergens (ng/g)
G01	Negative control	73.4	ND	ND	ND	ND	ND	ND
G02	Positive control	617.4	361.7	7,554.4	ND	18,521.0	96,623.0	122,698.4
G03	Comfort	35.6	ND	ND	ND	ND	ND	ND
G04	Contact	53.1	15.0	2,566.0	2,271.3	997.2	ND	5,834.5
G05	Derma Skin	22.0	1.4	96.4	ND	104.8	ND	201.2
G06	Micro-Thin Nutex	35.1	ND	471.3	488.6	ND	ND	959.9
G07	Micro-Touch	28.6	1.8	72.4	ND	ND	246.0	318.4
G08	Biogel Super Sensitive	11.9	1.2	43.3	ND	ND	55.4	98.7
G09/1	Biogel Eclipse Indicator	22.1	1.0	65.2	ND	ND	88.5	153.7
G09/2	Biogel Eclipse Indicator	19.9	1.6	238.2	ND	ND	197.0	435.2
G10	Peha-soft unsterile	ND	ND	229.5	ND	34.2	ND	263.8
G11	Peha-soft steril	ND	0.9	221.5	ND	50.2	ND	271.7
G12	Peha-micron plus	32.8	11.3	917.6	688.0	979.5	442.8	3,027.9
G13	Peha-taft plus	13.4	2.7	125.3	63.1	78.8	74.1	341.3
G14	Gentle Skin classic	ND	ND	ND	ND	ND	ND	ND
G15	Gentle Skin Anatom	7.1	ND	ND	ND	ND	ND	ND
G16	Gentle Skin grip	92.3	11.6	950.3	177.5	580.6	1,936.8	3,645.2
G17	Augustus polymer	ND	1.3	54.0	ND	68.7	ND	122.8
G18	Augustus puderfrei	17.1	2.1	41.3	ND	346.9	ND	388.2
G19	Augustus-Gel	ND	ND	ND	ND	39.1	ND	39.1

Given are the mean values of the single measurements. The determination of the protein content is based on 4 single measurements, that of the latex allergen and the Hev b 1 contents as double measurements, and the determination of the single allergens Hev b 3, 5, and 6.02 as a single measurement. The negative control (G01) could only be extracted with the IPA method and not with the EN method. If all single measurements were below the detection limit, the content is indicated by ND (not detectable).

**Table 2.** Protein, latex allergen, and single allergen contents of products of daily use (PDU) after extraction according to the IPA method.

Product	Name	Brand	Protein content (µg/g)	Latex allergen content (µg/g)	Hev b 1 content (ng/g)	Hev b 3 content (ng/g)	Hev b 5 content (ng/g)	Hev b 6.02 content (ng/g)	Sum of single allergens (ng/g)
PDU01	Pacifier	babylove	121.7	ND	1,986.8	ND	ND	ND	1,986.8
PDU02	Pacifier	nip	127.4	1.4	1,649.7	ND	ND	ND	1,649.7
PDU03	Pacifier	NUK	15.3	ND	478.9	ND	ND	ND	478.9
PDU04	Baby bottle nipple anti-colic	babylove	34.6	ND	362.0	ND	ND	ND	362.0
PDU05	Baby bottle nipple anti-colic	nip	49.4	ND	830.1	ND	ND	ND	830.1
PDU06	Baby bottle nipple anti-colic	NUK	35.0	ND	639.9	ND	ND	ND	639.9
PDU07	Condom	Billy Boy extra feucht	92.7	2.7	6,085.3	193.5	ND	93.0	6,371.8
PDU08	Condom	chaps classic natur	110.0	1.6	14,347.6	819.9	ND	260.9	15,428.4
PDU09	Condom	Durex love	ND	ND	1,489.1	ND	ND	ND	1,489.1
PDU10	Condom	Ritex Intensiv	202.9	1.4	9,987.9	ND	53.0	319.4	10,360.4
PDU11	Toy balloon	Adic B.V.	101.8	21.5	1,033.6	ND	3,124.9	7,726.1	11,884.7
PDU12	Disposable glove	Profissimo	44.7	4.4	ND	ND	1,065.2	572.5	1,637.7
PDU13	Household glove with cotton flocking	Profissimo	145.6	2.2	241.6	ND	172.3	399.1	813.0
PDU14	Band-Aid, elastic	Das gesunde Plus	150.3	2.5	ND	ND	ND	544.3	544.3

The determination of the protein and latex allergen contents as well as the determination of single allergens (Hev b 1, 3, 5, and 6.02) is based on one single measurement. If this single measurement was below the detection limit, the content is indicated by ND (not detectable).

**Table 3.** Comparison of protein, latex allergen, and Hev b 1 contents in untreated and boiled baby products.

Product	Name	Brand	Protein content (µg/g)		Reduction of protein content (%)	Latex allergen content (µg/g)		Hev b 1 content (ng/g)		Reduction of Hev b 1 content (%)
			untreated	boiled		untreated	boiled	untreated	boiled	
PDU01	Pacifier	babylove	121.7	49.5	59	ND	ND	1,986.8	496.1	75
PDU02	Pacifier	nip	127.4	35.7	72	1.4	ND	1,649.7	354.7	79
PDU03	Pacifier	NUK	15.3	ND	100	ND	ND	478.9	346.9	28
PDU04	Baby bottle nipple anti-colic	babylove	34.6	ND	100	ND	ND	362.0	302.8	16
PDU05	Baby bottle nipple anti-colic	nip	49.4	14.5	71	ND	ND	830.1	327.9	60
PDU06	Baby bottle nipple anti-colic	NUK	35.0	ND	100	ND	ND	639.9	295.2	54

The determination of the protein, latex allergen, and Hev b 1 contents are based on one single measurement. If this single measurement was below the detection limit, the content is indicated by ND (not detectable).
